# Propranolol in Treatment of Huge and Complicated Infantile Hemangiomas in Egyptian Children

**DOI:** 10.1155/2014/541810

**Published:** 2014-05-08

**Authors:** Basheir A. Hassan, Khalid S. Shreef

**Affiliations:** ^1^Pediatrics Department, Faculty of Medicine, Zagazig University, Zagazig 44519, Egypt; ^2^Surgery Department, Faculty of Medicine, Zagazig University, Zagazig 44519, Egypt

## Abstract

*Background*. Infantile hemangiomas (IHs) are the most common benign tumours of infancy. Propranolol has recently been reported to be a highly effective treatment for IHs. This study aimed to evaluate the efficacy and side effects of propranolol for treatment of complicated cases of IHs. *Patients and Methods*. This prospective clinical study included 30 children with huge or complicated IHs; their ages ranged from 2 months to 1 year. They were treated by oral propranolol. Treatment outcomes were clinically evaluated. *Results*. Superficial cutaneous hemangiomas began to respond to propranolol therapy within one to two weeks after the onset of treatment. The mean treatment period that was needed for the occurrence of complete resolution was 9.4 months. Treatment with propranolol was well tolerated and had few side effects. No rebound growth of the tumors was noted when propranolol dosing stopped except in one case. *Conclusion*. Propranolol is a promising treatment for IHs without obvious side effects. However, further studies with longer follow-up periods are needed.

## 1. Introduction

Infantile hemangiomas (IHs) are the most common benign tumors of infancy affecting 1% to 2.6% of newborns and up to 10% of children in the age of one. The incidence is higher in girls and premature infants. The majority of lesions involve the skin and subcutaneous tissues and eventually resolve spontaneously without complications [1–4].

However, about 20% of IHs are extremely disfiguring and destructive to normal tissue and may even be life threatening [5]. A central characteristic of most IHs is a predictable life cycle. The initial proliferative phase lasts 6 to 10 months, during which the tumor grows rapidly because of excessive vascular endothelial cells (ECs). This is followed by an involution phase, spanning up to 7 years. Normal involution is thought to occur via cell senility and eventual apoptosis [5–7].

Ulceration is the most common complication and occurs most often toward the end of the growth phase [8]. However, in some IHs, ulceration precedes proliferation and is actually the presenting clinical sign of IHs [8, 9]. This early ulceration suggests that it may not simply be a response to rapid growth or tensile stress on the overlying skin but other causes such as local tissue hypoxia may play a role [10, 11].

Lesions can be subtyped as localized (which originate from a focal point), segmental (which follow a geographic distribution), indeterminate, and multifocal (8 or more noncontiguous lesions) [12].

Approximately 80% of IHs are found on the face and neck, favoring the anterior cheek, forehead, and preauricular area [5, 12]. The remaining IHs are found in decreasing order on the trunk, lower extremities, and upper extremities and occasionally on mucosal surfaces.

Facial IHs were divided according to embryological segments as suggested by Haggstrom et al. [13] as follows: frontonasal, frontotemporal, maxillary, and mandibular.

Corticosteroids, interferon-alfa, vincristine, laser therapy, and topical imiquimod are used in the treatment of IHs [5]. Surgical excision had also been reported to be effective.

Recently, the use of propranolol, a nonselective beta blocker, has been described in the treatment of IHs, with a spectacular efficacy in all cases [3]. Proposed mechanisms of the action of propranolol on hemangiomas include control of hypoxic stress with upregulated HIF-1a, apoptosis induction, and decreased production of endothelial vascular and fibroblastic growth factors [3].

So, our study aimed to describe the efficacy of propranolol in 30 children with IHs and the adverse effects related to this therapy.

## 2. Patients and Methods

This prospective clinical study was carried out at the Pediatric Department and Pediatric Surgery Unit, Zagazig University Hospitals, Egypt, during the period from April 2010 to September 2012.

The patients were included in the study according to the following inclusion criteria: eyelid involvement with ocular risk of occlusion or compression, airway obstruction, large IHs with considerable aesthetic derangement or ulceration, and all rapidly proliferating hemangiomas with functional deficit and/or disfigurement.

Exclusion criteria included history of hypoglycemia, asthma or bronchospasm, patients with known cardiac abnormalities, and patients with PHACES (posterior fossa malformations, hemangioma, arterial anomalies, cardiac defects, eye abnormalities, and sternal clefts) syndrome.

All infants were subjected to full history taking and thorough clinical examination which included complete cardiac examination, electrocardiography, and echocardiography. As most of our patients included in this study had segmental IHs in the face, a cerebral magnetic resonance imaging/magnetic resonance angiography (MRI/MRA), electrocardiography, and echocardiography were carried out in these patients before initiating therapy to rule out PHACES syndrome.

All patients were admitted to hospital during the first two days of treatment for monitoring of blood pressure, heart rate, and serum glucose level one to three times a day; measurements were taken before and 2 hours after each dose of propranolol. At the first week, the dose of propranolol was 1 mg/kg/day and then increased to full dosage (1.5 mg/kg/12 hours). The full dosage was maintained during the whole period of the study. A treatment period of at least 12 weeks was scheduled, which could be further expanded if the patient required more time to achieve the resolution of the problem that had led to initiation of propranolol therapy. Propranolol was withdrawn gradually over a period of at least 4 weeks.

Follow-up visits were scheduled every 2 weeks in the outpatient clinic for further physical examination of IH and monitoring of ECG, blood pressure, and heart rate. During the treatment period, the progressive effect of propranolol therapy was measured by comparing the diameters and colors in the front and lateral photos of every patient that were taken before treatment and at every follow-up visit.

Informed written consents for propranolol treatment and use of the patients' photographs were obtained prior to inclusion in the study from the children's guardians. The study protocol was approved by the Pediatric Ethical Committee in Zagazig University.

## 3. Results

Thirty patients (21 females, 9 males) (their ages ranged from 2 months to 1 year) with different sites of IHs were treated with propranolol. Demographic data, types, locations of hemangiomas, duration, and side effects of treatment of these patients are summarized in Table 1.

The mean age for starting propranolol treatment was 3.7 months (ranged from 2 to 12 months). IHs were seen more commonly in preterm than full-term babies (73.3% versus 26.6%) with female predominance (70%).

The appearance of hemangiomas occurred after the age of one month in 23 (76.6%) patients. Eighteen (60%) patients had localized hemangiomas; 9 (30%) patients had segmental lesions while 3 (10%) patients had multifocal lesion.

The anatomical sites of hemangiomas were shown in Table 1.

Twelve (40%) patients had impairment of vision, hearing, and/or difficult swallowing. Three (10%) patients had painful ulceration in the face and two (6.6%) patients had disfiguring hemangioma in the face.

**Figure 1:**
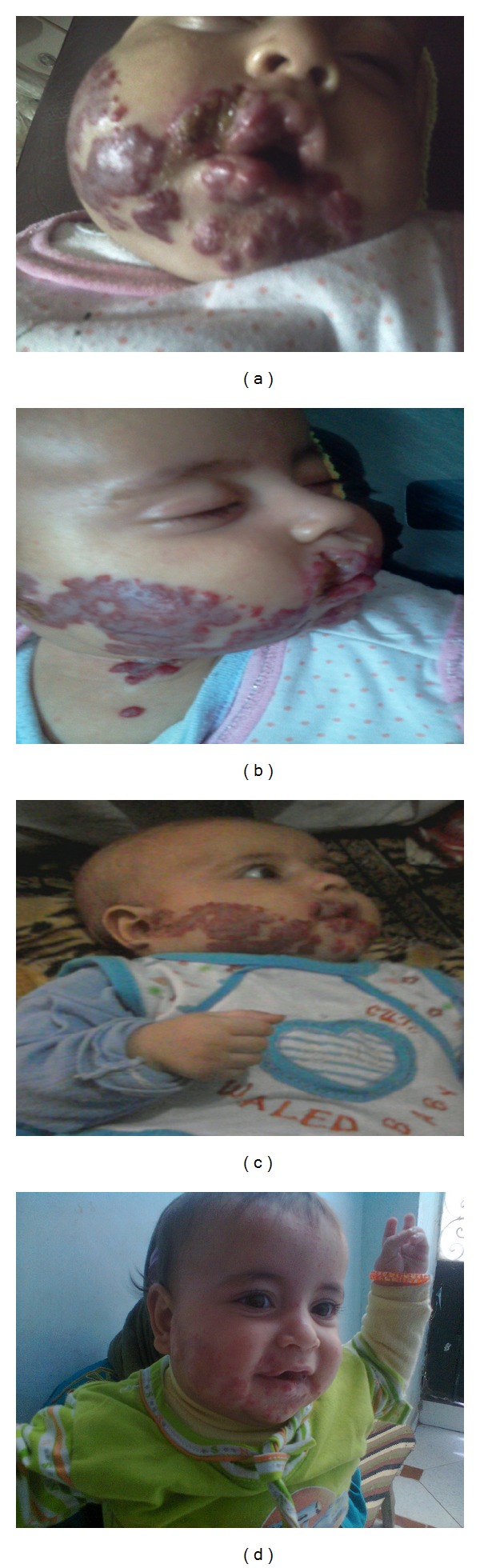
Hemangioma in patient number 1. Before treatment (a), one week (b), one month (c), and six months after treatment (d).

**Figure 2:**
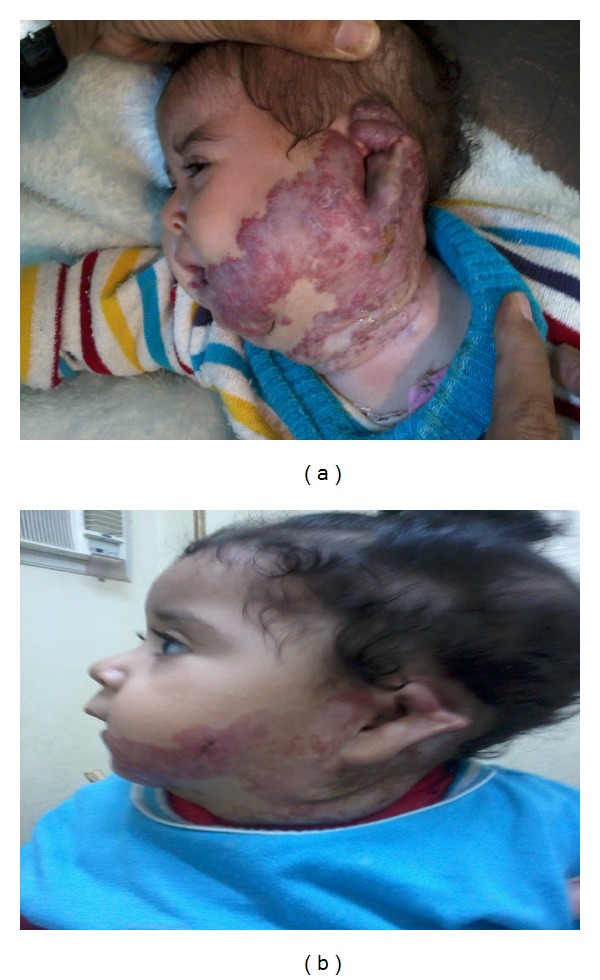
Hemangioma in patient number 10. Before treatment (a) and 8 months after treatment (b).

**Figure 3:**
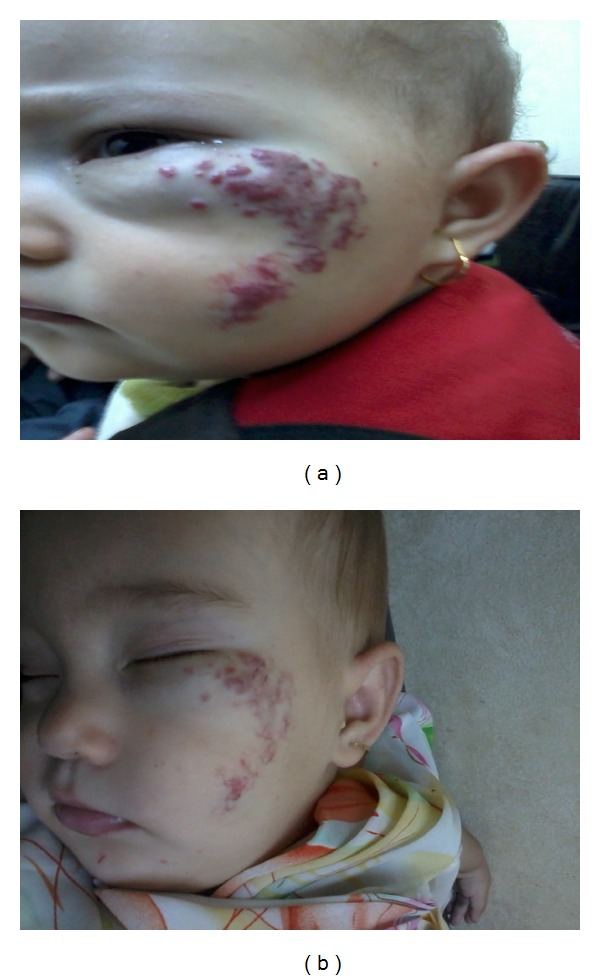
Hemangioma in patient number 11. Before treatment (a) and 8 months after treatment (b).

Superficial cutaneous hemangiomas started to respond to propranolol therapy within one to two weeks after the onset of treatment. They began to change from intense red to purple with softening and continued to improve until they became nearly flat with significantly diminished color, together with regression of the tumor size (Figures 1, 2, and 3).

The ulcerated IHs experienced a reduction in severity similar to nonulcerated hemangiomas, and ulceration was completely resolved in all patients within 2–8 weeks of treatment.

The treatment period that was needed for the occurrence of complete resolution ranged from 6 to 14 months (mean 9.4). After resolution, the treatment was withdrawn gradually over a period of at least 4 weeks.

Regarding propranolol-associated side effects, 2 (6.6%) patients had a wheezy chest and tachypnea at 2 weeks after treatment and one (3.3%) patient developed hypoglycemia at 1 month after treatment. Cold extremities were found in one (3.3%) patient and constipation developed in 2 patients (6.6%).

No rebound growth of the tumors was noted after the stoppage of treatment except in one case with complicated left retroorbital hemangioma with lagophthalmos and proptosis.

## 4. Discussion

Most hemangiomas are relatively small and pose only minor clinical problems, but approximately 20% become clinically significant and require treatment. This may be a result of their aggressive growth and/or their location close to vital structures that they can invade, impairing the function and thus threatening the child's life; large hemangiomas in the area of the mouth, nose, or eyes prevent normal feeding, respiration, or vision [1, 14].

Ulceration, hemorrhage, infection, and high output cardiac failure may complicate IHs. Even apart from such clinically problematic situations, hemangiomas are often disfiguring and can have significant psychological/emotional impact on the affected child. This causes many parents to seek treatment rather than wait for the natural involution to occur [15].

Propranolol hydrochloride has been used in children for a variety of disorders, but its effectiveness in the treatment of IHs was only recently discovered [3]. So we designed this study to evaluate the efficacy of propranolol in the treatment of huge and/or complicated types of IHs and to assess side effects related to this therapy.

In our study, there was a predominance of female and premature infants among the patients. These results were consistent with that of Hsu et al.'s study [16].

In our study, the mean duration of therapy was 9.4 months. In the literature, duration of therapy varies from 2 to 10 months. Hogeling and his colleagues stated that IHs significantly dropped in volume, redness, and elevation from the skin with a six-month course of propranolol [17]. Bertrand et al., in their study, stated that all patients treated with propranolol had good to excellent improvement at 6 months and the mean duration of treatment was 10.6 months (range 7–13 months) [18].

The drug appeared to be safe with no significant hypotension, hypoglycemia, or bradycardia among our patients. This efficacy and safety have been observed in other studies as demonstrated in a recent systematic literature review summarizing the effectiveness and adverse effects of propranolol in the treatment of IHs [19]. Lawley et al. reported two cases who received propranolol in the recommended dosage; one experienced severe hypotension and the other experienced severe hypoglycemia [20]. Other authors have reported similar cases [21, 22]. Buckmiller [23] observed gastroesophageal reflux and somnolence in some patients. Adverse effects of propranolol have also been reported, including hypotension, bradycardia, bronchospasm, and hypoglycemia [24].

In conclusion, this study contributes to the growing evidence that oral propranolol is an efficacious and safe treatment for IHs, with a careful dosing and monitoring regimen. Nevertheless, further studies and longer followup are needed.

## Figures and Tables

**Table 1:** Demographic and clinical data of study population variable.

Total number of patients	**30** (**100%**)
Preterm	**22** (**73.3%**)
Fullterm	**8** (**26.6%**)
Age at initiation of treatment (month),	
Mean ± SD	3.7 ± 2.5
Gender, *n* (%)	
Male	**9** (30%)
Female	**21** (70%)
Type, *n* (%)	
Localized	**18** (60%)
Segmental	**9** (30%)
Multifocal	**3** (**10%**)
Location of IH, *n* (%)	
Facial Segments:	
Frontotemporal (seg.1)	3 (10%)
Maxillary (seg.2)	5 (16.6%)
Mandibular (seg.3)	8 (26.6%)
Frontonasal (seg.4)	2 (6.6%)
Other locations:	
Trunk	8 (26.6%)
Extremities	3 (10%)
Neck	1 (3.3%)
Duration of treatment till resolution (month),	9.4 ± 2.6
Mean (range)	(6–14)
Side effects of treatment, *n* (%)	
Tachypnea	**2** (6.6%)
Hypoglycemia	**1** (3.3%)
Cold extrmities	**1** (3.3%)
Constipation	**2** (6.6%)
